# Winter Survival of Individual Honey Bees and Honey Bee Colonies Depends on Level of *Varroa destructor* Infestation

**DOI:** 10.1371/journal.pone.0036285

**Published:** 2012-04-27

**Authors:** Coby van Dooremalen, Lonne Gerritsen, Bram Cornelissen, Jozef J. M. van der Steen, Frank van Langevelde, Tjeerd Blacquière

**Affiliations:** 1 Bees@wur, Plant Research International, Wageningen, The Netherlands; 2 Resource Ecology Group, Wageningen University, Wageningen, The Netherlands; United States Department of Agriculture, Agriculture Research Service, United States of America

## Abstract

**Background:**

Recent elevated winter loss of honey bee colonies is a major concern. The presence of the mite *Varroa destructor* in colonies places an important pressure on bee health. *V. destructor* shortens the lifespan of individual bees, while long lifespan during winter is a primary requirement to survive until the next spring. We investigated in two subsequent years the effects of different levels of *V. destructor* infestation during the transition from short-lived summer bees to long-lived winter bees on the lifespan of individual bees and the survival of bee colonies during winter. Colonies treated earlier in the season to reduce *V. destructor* infestation during the development of winter bees were expected to have longer bee lifespan and higher colony survival after winter.

**Methodology/Principal Findings:**

Mite infestation was reduced using acaricide treatments during different months (July, August, September, or not treated). We found that the number of capped brood cells decreased drastically between August and November, while at the same time, the lifespan of the bees (marked cohorts) increased indicating the transition to winter bees. Low *V. destructor* infestation levels before and during the transition to winter bees resulted in an increase in lifespan of bees and higher colony survival compared to colonies that were not treated and that had higher infestation levels. A variety of stress-related factors could have contributed to the variation in longevity and winter survival that we found between years.

**Conclusions/Significance:**

This study contributes to theory about the multiple causes for the recent elevated colony losses in honey bees. Our study shows the correlation between long lifespan of winter bees and colony loss in spring. Moreover, we show that colonies treated earlier in the season had reduced *V. destructor* infestation during the development of winter bees resulting in longer bee lifespan and higher colony survival after winter.

## Introduction

The parasitic mite *Varroa destructor* is considered to be one of the main causes for colony losses in honey bees (*Apis mellifera* L.) [1]–[4]. For example, the total number of honey producing colonies in the U.S. was reduced by 1.5±0.7% (mean ± s.e.) per year since the introduction of *V. destructor*, while the decrease per year used to be on average 0.06±0.5% [1]. This decline reflects the loss of colonies as well as the decline in number of beekeepers due to increased expenses and efforts needed to combat mite infestations [1], [5]. Although there is a general agreement that there is no single explanation for the extensive colony losses, and that interactions between different stresses are likely to be involved, the presence of *V. destructor* in colonies places an important pressure on bee health [2]. *V. destructor* reduces the body weight and protein content of individual bees, which is found to shorten their lifespan [6]–[8]. This is especially important during winter in temperate regions when long lifespans are a primary requirement to survive until the next spring and to nurse the first brood [7], [8]. In the temperate regions, the main colony losses due to *V. destructor* occur during winter [8]. Nowadays, winter losses are often up to 20% or more in many areas [1], [3], while twenty years ago, 5 to 10% colony losses during winter were common [2].

**Figure 1:**
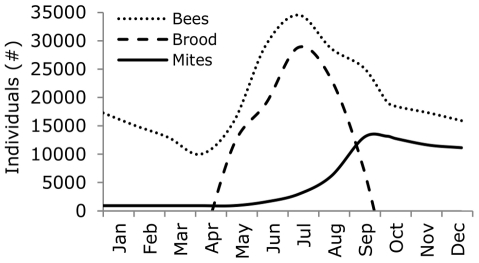
Colony development for adult bees, worker brood, and *Varroa destructor* mites. The daily number of individual adult bees (dotted line) and worker brood (striped line) was modelled over one year. The number of mites (solid line) was modelled as being the second year of mite infestation with a starting population of 100 mites in the first year. Figure was redrawn from Martin [9].

In temperate regions, the number of bees and brood in a colony increase between April and July and decrease between August and October [9]. However, the main peak of the number of bees and brood occur earlier in the season than the peak of mite abundance [10], [11]. Hence, mite infestation strongly increases during the period in which the number of bees and brood decrease [9] (Figure 1), resulting in an increasing number of brood cells infested with *V. destructor* over time. It is exactly during these months of reduction in the number of brood and rapid increase in mite infestation, that bees hatching from this highly infested brood will become winter bees [9], [12].

Adult bees, which are infested by *V. destructor* as pupae, do not fully develop physiological features typical of long-lived winter bees compared with non-infested workers [6]–[8], making it unlikely for them to survive until spring and contribute to the build-up of the colony in early spring [2]. To date, however, the relation between the lifespan of individual bees and colony losses for different levels of *V. destructor* infestation has not been tested.

When the European honey bee (*Apis mellifera*) was moved to areas where the Asian honey bee (*A. ceranae*) was endemic, *V. destructor* switched to *A. mellifera* and spread nearly worldwide [2], [4]. During the first years after its introduction in Europe and North America, *V. destructor* could be easily controlled and be kept below damaging infestation levels by one to two acaricide treatments per year. However, colony losses have recently started to increase drastically, despite the development of more intensive acaricide treatments [1], [2]. Absence, poor timing and poor application of acaricide treatment have been reported to be important causes for honey bee colony loss [13], [14]. Especially when honey is harvested at the end of the bee season in temperate regions, acaricide treatments are often postponed until after the harvest to avoid residues in honey. However, the mite population has often already reached injurious levels at this time, namely the time that winter bees are produced (Figure 1). Consequently, timing of acaricide treatment in the second half of the summer season (July to September) may thus affect winter survival of the colony.

**Figure 2:**
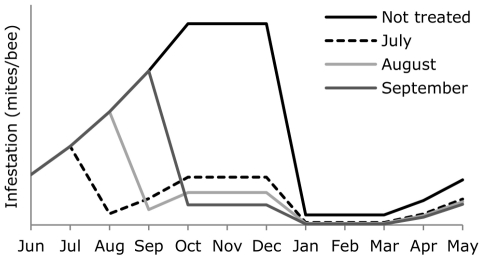
Expected infestation levels of *Varroa destructor* manipulated using acaricide treatment. Infestation levels of *V. destructor* (mites/bee) were manipulated using acaricide treatment applied at different moments (July, August, September or not treated at all). For the expected mite infestation, we used a simplified curve from mite infestation in Figure 1, with an exponential increase in mite infestation until October, after which the infestation remained equal. Efficacy of the acaricide Thymovar (July, August, September) was assumed to be 90%, while efficacy of oxalic acid (December) was assumed to be 95%.

In this study, the effect of different levels of *V. destructor* infestation during the transition from short-lived summer bees to long-lived winter bees on the lifespan of individual bees and the survival of bee colonies during winter was investigated. We manipulated the level of *V. destructor* infestation by reducing the number of mites using acaricide treatments at different times (during July, August, September, or not treated), resulting in increased mite fall directly during acaricide treatment and in reduced *V. destructor* infestation level in the months after this treatment (Figure 2 gives the expected infestation levels for different treatment moments). We expected a longer lifespan of bees in colonies treated earlier in the season, as low infestation levels during the development of winter bees should benefit the lifespan of these bees compared to colonies treated later in the year or not at all. Consequently, colonies with relatively low *V. destructor* infestation during the development of winter bees are expected to have higher colony survival during or after winter. The experiment was performed in two consecutive years as environmental conditions such as weather or food resources are expected to also affect winter bee development and colony survival.

## Materials and Methods

### Experiment

The fieldwork took place in 2005/2006 and 2006/2007 at an apiary of Wageningen UR, The Netherlands (51°59′32.35″N, 5°39′46.81″E). Colonies (N = 24) were kept in one-story wooden hives with 10 frames and contained brood in all developmental phases. In the first year (2005/2006), mite fall was monitored in the colonies for one week in July. The colonies with the lowest daily mite fall (2.9±0.78, N = 6) were used to represent the low *V. destructor* infestation from July onwards. The remaining colonies were randomly allocated to 3 groups: treated in August, treated in September, or not treated at all. The mean daily mite fall in these groups did not differ from each other (overall mean daily mite fall 18.8±3.5, N = 18), but were all higher compared to the colonies with low infestation from July onwards (daily mite fall was Log10-transformed, Anova, F_3,20_ = 9.31, P<0.001, Sidak post hoc test). In the second year (2006/2007), colonies were randomly allocated to 4 groups: treated in July, treated in August, treated in September, or not treated at all. Colonies were treated with the acaricide Thymovar® during three weeks in the allocated month. The experiment in the second year was performed with new colonies. Colonies that became queenless or swarmed were removed from the study.

Daily mite fall in debris was monitored to give an indicative efficacy of the Thymovar® treatment during and after the treatment periods, starting in August. Outside these periods, mite fall was counted once a week (trapping period of 4 days with a bottom board) to get an indication of the infestation level. In winter (November/December), when there was no more brood, all colonies were treated with an oxalic acid solution (trickling, 37 gr oxalic acid dihydrate in 1 L sugar water, 1∶1 weight ratio for sucrose: water). Mite fall was counted after trapping for one week continuously following the oxalic acid treatment. Thereafter, mite fall was monitored every two weeks (trapping period of 2 days). Counting mite fall has been shown to be effective to estimate the population of mites [15], [16].

In half of the colonies of each experimental group, the number of capped brood cells was estimated by superimposing a grid with 2.5×2.5 cm squares over the brood area. Solid squares were counted directly and partial squares estimated. The number of brood cells was then calculated from the number of grids multiplied by 25 brood cells (we counted 400 cells in one dm^2^). Brood was measured every two weeks from mid-August until mid-November. During 2006/2007, due to the high winter temperatures, brood measurements were continued every month until mid-April.

Every fortnight, cohorts of approximately 100 newly emerged bees were marked with a unique colour (colour marker Posca) and returned to their original colony. In 2005 marking cohorts started in July, resulting in eight cohorts in four (out of six) colonies per treatment. In 2006 marking started in August, resulting in seven cohorts in four (out of six) colonies per treatment. Marking cohorts was stopped at the beginning of November in both years. At equal intervals, the presence of bees from previously marked cohorts was recorded. Based on the unique colour the age of the bees could be determined. Recording cohort survival continued until mid-April the following year or until no more marked bees were observed. If colonies could not be examined during winter, it was assumed that worker mortality was constant.

After winter in April, the size of the colony was estimated by counting the number of frames with bees. Non-surviving colonies had zero frames with bees.

### Statistics

To test whether the weather differed between the two years, the differences in ambient temperature were tested with a paired t-test (paired for month) for the period July–November and the period December–April separately in 2005/2006 and 2006/2007.

Mean daily mite fall per colony was calculated per month. Repeated measures ANOVAs were used to test mite fall for 2005/2006 and 2006/2007 separately, as mean daily mite fall in one month was assumed to be correlated to mean daily mite fall in the previous month. Sidak posthoc tests were used for pair wise comparison of differences between means. In 2005/2006, one colony from the group treated in September was excluded from the analysis due to missing data on mite fall for several months. One colony (treated in September) missed data on mite fall only in August. We interpolated this missing data in August using data from another colony, which was selected based on similar mite fall in September. Two colonies of the group that was not treated lacked data in March and April due to mortality of these colonies. To be able to use the Repeated measures ANOVA, we estimated the mite fall in these colonies to be similar to the highest mite fall found in the months March and April for all treatments. Slight changes in the estimated mite fall (approx. 10%) did not qualitatively change the results. Final number of colonies used in the Repeated measures ANOVA for mite fall in 2005/2006 were 6 (treated in July), 7 (treated in August), 5 (treated in September), and 5 (not treated).

In 2006/2007, in total 11 colonies were excluded from the analysis for mite fall due to missing data on mite fall for several months: two colonies in the group treated in July, two in the group treated in August, one in the group treated in September, and eight in the group that was not treated. All these excluded colonies were lost between October and November 2006, possibly due to high *V. destructor* infestation. To test whether excluded colonies showed higher mite fall until October than the remaining colonies, we used the Repeated measures ANOVA for mite fall in August to October, for excluded colonies (N = 11) and colonies still in the experiment (N = 24). Final number of experimental colonies used in the Repeated measures ANOVA for mite fall in 2006/2007 was 7 (treated in July), 6 (August), 6 (September), and 5 (not treated).

We calculated the survival rate for each cohort of bees marked in a colony, using survival analysis with Cox Proportional Hazards Models for treatment (timing of acaricide application), for the number of days since the cohorts were marked (is equal to the day the bees in that cohort were born), and for 2005/2006 and 2006/2007 separately. In addition to testing the differences between treatments and the differences over time, the Cox Proportional Hazards Model was used to predict the fraction of bees in a cohort that is still alive (in statistical terms this is called the predicted survival probability) at a certain age of the cohort, from here onwards called ‘bee survival’. To compare treatments over time, we calculated bee survival at 100 days (fraction of bees still alive at the age of 100 days). As summer bees only live for about 35 days, while winter bees live for about 135 days [12] or 150 days [17], we assume that bees that are alive after 100 days are winter bees. Low bee survival at 100 days means that the mean lifespan of the bees in the cohort is short. Consequently this means for winter bees that fewer bees will survive until spring and be able to contribute to spring development of the colony. To test whether bee survival at 100 days differed between 2005/2006 and 2006/2007, Repeated measures ANOVA was used. As the days the cohorts were marked did not coincide perfectly between the years, we paired the days most similar for both years (maximum difference was 2 days) and excluded the cohorts marked in July 2005 (no cohorts were marked in July 2006). Additionally, as we had only one mean value per day of marking per treatment, the treatments were pooled (N = 4 per day of marking).

Repeated measures ANOVAs were used to analyse the change in the number of capped brood cells over time in 2005/2006 and 2006/2007 separately. When colonies were lost during the experiment, brood measurements of other colonies within the experiment were used to continue the brood measurements. In 2005/2006, capped brood cells were counted from August to November and in April. Between November 2005 and April 2006, actual counts of capped brood cells were suspended due to cold temperatures and dense clustering of bees. The final number of colonies for counting capped brood cells was 3 for colonies treated in July, 3 for colonies treated in August, 2 for colonies treated in September, and 3 for colonies that were not treated. In 2006/2007, brood cells were counted continuously from August to April. The final number of colonies for counting capped brood cells was 4 for colonies treated in July, 3 for colonies treated in August, 3 for colonies treated in September, and 3 for colonies that were not treated.

To test whether the fraction of winter bees in a cohort increased during the decrease of brood in autumn we used a General Linear Model. Mean bee survival at 100 days of the different treatments (timing of acaricide application, fixed factor) was tested as a function of the number of brood cells (as covariate) for 2005/2006 and 2006/2007 separately. Possible interactions between treatments in relation to the decrease in brood were added to show differences in the rate of change in bee survival. Sidak posthoc tests for pair wise comparison were used to test for differences between treatments.

To test the differences in colony size in April between the treatments, we calculated the mean fraction of frames that was occupied with bees in April using a Generalized Linear Model. If a colony had died before April, the number of frames occupied by bees was zero. The mean fraction of frames was estimated with the number of occupied frames in April as dependent variable and the 10 frames that were available in each hive as fixed number of trials (binomial distribution and logit link function). Sidak posthoc tests for pair wise comparison were used to test differences in the mean fraction of frames between treatments. A Pearson correlation was used to test if there was a correlation between bee survival at 100 days for the cohort that was marked (born) in November and the fraction of frames occupied with bees in April.

## Results

### Ambient temperature

The mean ambient temperature during summer and autumn (July–November) in The Netherlands did not differ between 2005 (14.0±1.9°C) and 2006 (15.9±2.2°C; paired t-test: t_4_ = −2.38, P = 0.08). Mean temperature between December 2005 to April 2006 was however lower (4.3±1.3°C) than between December 2006 and April 2007 (8.1±1.3°C; paired t-test: t_4_ = −7.35, P<0.01).

### Acaricide treatment effectiveness (mite fall)

**Figure 3:**
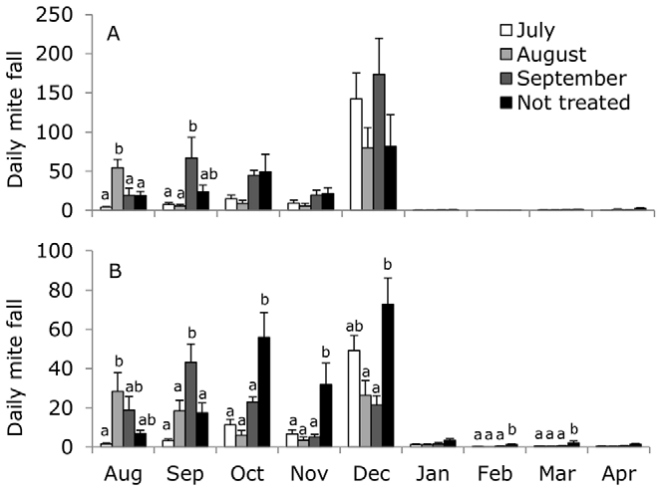
Mean daily mite fall in 2005/2006 (A) and 2006/2007 (B). Colonies were treated with Thymovar® in July (white bars), August (grey bars), September (dark grey bars), or not treated at all (black bars). All colonies were treated in December using oxalic acid (3%). Letters denote significant differences between treatments within each month. No letters mean no significant differences between treatments were found. Differences between months were not given.

In 2005/2006, mean daily mite fall differed between the treatments per month (Repeated measures ANOVA: treatment F_3,19_ = 2.76, P = 0.07; month F_8,152_ = 32.87, P<0.001; treatment×month F_24,152_ = 2,39, P = 0.001; Figure 3A). As can be expected, mean daily mite fall in August was highest for colonies treated in August, and highest in September for colonies treated in September. In 2006/2007, mean daily mite fall also differed between the treatments per month (Repeated measures ANOVA: treatment F_3,20_ = 7.63, P = 0.001; month F_8,160_ = 41.38, P<0.001; treatment×month F_24,152_ = 8.17, P<0.001; Figure 3B). Again, daily mite fall in August was highest for colonies treated in August, and highest in September for colonies treated in September. Daily mite fall for colonies that were not treated remained high during the year. In 2006, colonies that were lost between October and November and excluded from the analysis above indeed showed higher daily mite fall (overall 38.7±4.9) than colonies included in the analysis (overall 18.6±3.3), where the daily mite fall increased with time (month), but more for the colonies excluded than for colonies included in the analysis (Repeated measures ANOVA: in/excluded F_1,33_ = 11.33, P = 0.002; month F_1,33_ = 13.82, P = 0.001; in/excluded×month F_1,33_ = 5.59, P = 0.02).

### Bee survival

**Figure 4:**
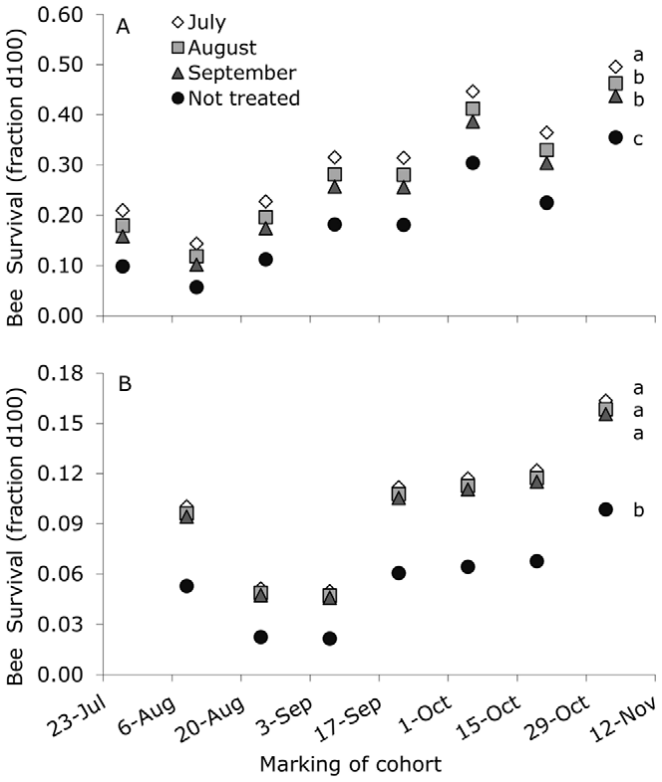
Bee survival as a function of time and treatment in 2005/2006 (A) and 2006/2007 (B). Bee survival (fraction d100) was the predicted fraction of bees that was still alive at the age of 100 days, and calculated using a Cox Proportional Hazards Model. Time was the marking date of the cohort (scatterplot). Different months of acaricide application show the treatments, where letters denote significant differences (over all marking dates).

Mean survivorship curves for marked cohorts of bees are shown in Figure S1. For the survival analysis, the Cox Proportional Hazards Models used 6398 uncensored cases and 353 censored cases for 2005/2006, and 8458 uncensored cases and 600 censored cases for 2006/2007. The cumulative survival curves for the different treatments over time clearly showed a lower bee survival in colonies that were not treated compared to all other treatments in both 2005/2006 (Wald = 123.2, df = 3, P<0.001) and 2006/2007 (Wald = 87.2, df = 3, P<0.001; Figure S2). We found that the cumulative survival increased with time in both 2005/2006 (Wald = 435.1, df = 6, P<0.001) and 2006/2007 (Wald = 200.4, df = 6, P<0.001; Figure S2), suggesting an increasing fraction of winter bees in the cohorts. Bee survival at day 100 (fraction of bees still alive at the age of 100 days) was predicted by the model as a function of time and treatment in 2005/2006 and 2006/2007 (Figure 4). For both 2005/2006 and 2006/2007, using day 50, 75 or 120 did not qualitatively change the results. When marked in 2005, bee survival at 100 days was higher than in 2006 from August 24^th^ onwards (2006, coinciding with August 25^th^ for 2005), and this difference became larger towards the end (Repeated measures ANOVA: year F_1,21_ = 805.70, P<0.001; marking day F_6,21_ = 13.83, P<0.001; year×marking day F_6,21_ = 29.31, P<0.001). For example, from bees that emerged on November 4^th^ 2005 44±3% was still alive at an age of 100 days, while from bees that emerged on November 2^nd^ 2006 only 14±1% was still alive at an age of 100 days.

### Colony development (brood)

**Figure 5:**
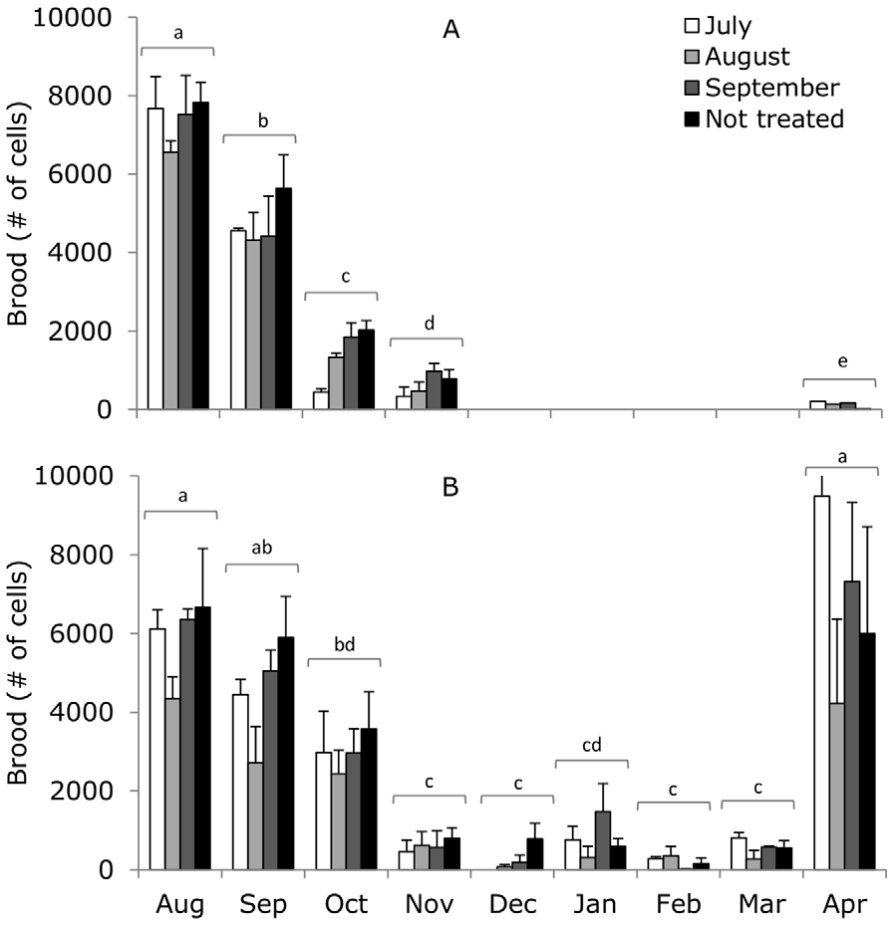
Mean number of capped brood cells in 2005/2006 (A) and 2006/2007 (B). Colonies were treated with Thymovar® in July (white bars), August (grey bars), September (dark grey bars), or not treated at all (black bars). Number of capped brood cells between December 2005 and March 2006 were not measured due to cold winter temperatures. Letters show significant differences between months.

In 2005/2006, the number of capped brood cells decreased between August and November (Repeated measures ANOVA: treatment F_3,7_ = 1.59, P = 0.28; month F_4,28_ = 223.65, P<0.001; treatment×month F_12,28_ = 1.20, P = 0.33; Figure 5A). Brood rearing had not yet shown the expected spring increase in April 2006 for any of the treatments. In 2006/2007, the number of capped brood cells also decreased between August and November (Repeated measures ANOVA: treatment F_3,9_ = 3.89, P = 0.05; month F_8,72_ = 38.25, P<0.001; treatment×month F_24,72_ = 1.20, P = 0.38; Figure 5B). Brood rearing continued at a low rate during winter and was much increased in April 2007 for all treatments. Although the Repeated measures ANOVA showed a borderline significant effect of treatment for 2006/2007, the Sidak posthoc test did not show differences between treatments (the number of capped brood cells for colonies treated in July was almost higher than brood for colonies treated in August, Sidak P = 0.08).

### Bee survival in relation to number of capped brood cells

**Figure 6:**
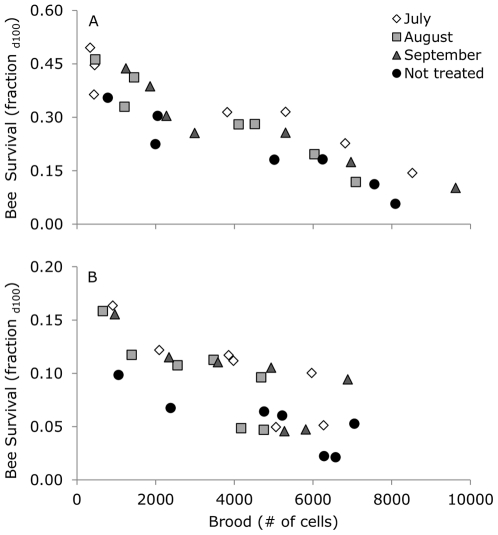
Bee survival as a function of brood in 2005/2006 (A) and 2006/2007 (B). Bee survival (fraction d100), the predicted fraction of bees that was still alive at the age of 100 days, as function of the number of capped brood cells for the different months of acaricide application. Symbols show means per marking day.

In 2005/2006, bee survival increased with a decrease in number of capped brood cells (General Linear Model: treatment F_3,23_ = 6.52, P<0.01; brood F_1,23_ = 162.39, P<0.001; Figure 6A; if the interaction was included, then both the interaction between treatment×brood and the main effect treatment were not significant). In relation to brood, there was a lower bee survival for colonies that were not treated than for colonies treated in July or September, but not lower than colonies treated in August. In 2006/2007, bee survival also increased with a decrease in number of capped brood cells (General Linear Model: treatment F_3,23_ = 3.60, P<0.05; brood F_1,23_ = 38.59, P<0.001; Figure 6B; if the interaction was included, then both the interaction between treatment×brood and the main effect treatment were not significant). There was a lower bee survival for colonies that were not treated than for colonies treated in July, but not compared to colonies treated in August or September.

### Winter survival

**Figure 7:**
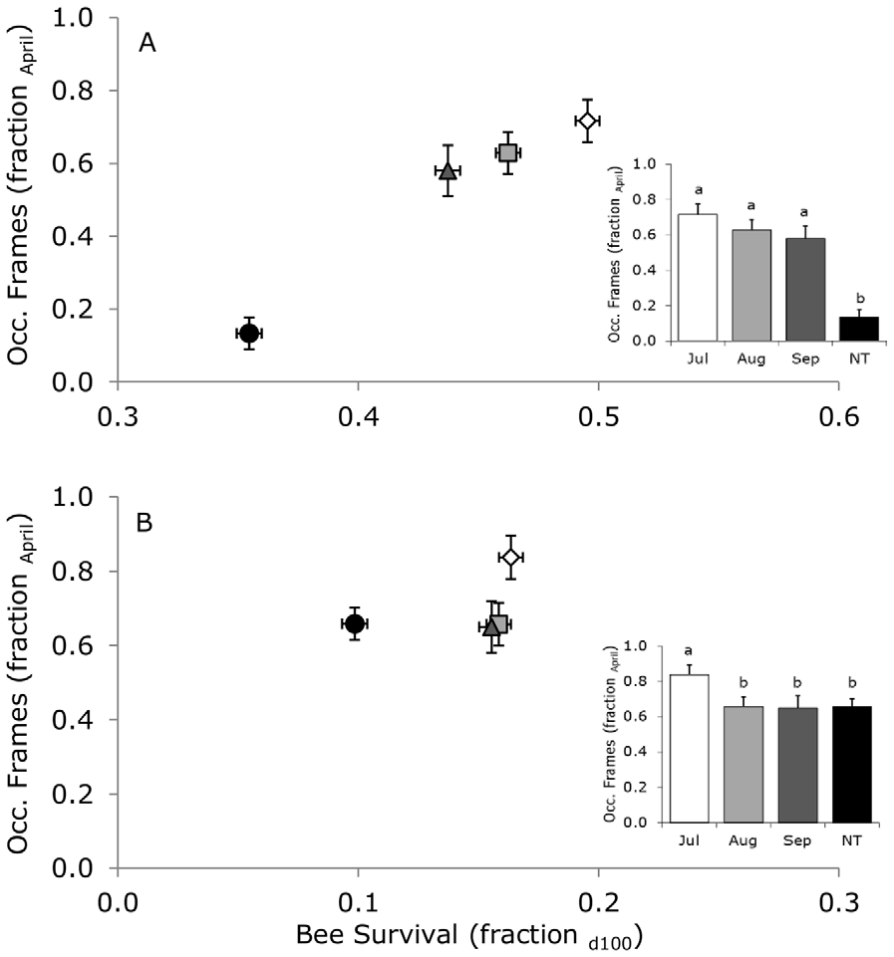
Winter survival as a function of bee survival in November 2005 (B) and 2006 (B). Fraction of frames occupied with bees in a colony in April in relation to bee survival at 100 days for the cohorts marked in November 2005 (A) and November 2006 (B). We used the data for November as an example, the relationship is similar for all days of marking, the trend only showed lower bee survival for cohorts marked earlier. Inserts show the differences in the fraction of frames occupied between for the different treatments (timing of acaricide application, NT = not treated). Letters indicate significant differences.

Between November 2005 and April 2006, four colonies were lost in the group not treated with acaricide, while no winter colony loss occurred in the other groups. The fraction of frames (out of 10) that were occupied with bees in April 2006 was the lowest for colonies that were not treated in 2005 (Generalized Linear Model: Wald Chi-Square = 38.1, df = 3, P<0.001; Figure 7A insert), and increased with an increase in bee survival (Pearson correlation: r = 0.98, n = 4, P = 0.02; Figure 7A). During the winter of 2006/2007, no colonies were lost. The fraction of frames (out of 10) that was occupied with bees in April 2007 was highest for the colonies that were treated with acaricide in July 2006 (Generalized Linear Model: Wald Chi-Square = 9.2, df = 3, P = 0.027; Figure 7B insert), but did not relate to bee survival (Pearson correlation: r = 0.42, n = 4, P = 0.58; Figure 7B). For the relation between the fraction of frames that were occupied with bees in April and bee survival, data from November was used as an example: the relationships were similar for all days the cohorts were marked, the trend only showed lower bee survival for cohorts marked earlier.

## Discussion

In this study, we found that low *V. destructor* infestation levels during the development of winter bees resulted in an increase in lifespan of bees compared to colonies that were not treated and that had higher infestation levels. Acaricide treatment before the expected transition period from summer to winter bees resulted in the highest lifespan of bees. Colonies with low infestation levels had fewer losses in number of bees and higher survival during and after winter. A large number of bees in the bee colony at the start of the growing season in temperate regions has indeed shown to increase survival and production of bee colonies [18]. Several studies reported the decrease in lifespan of individual bees due to *V. destructor* infestation [6], [7], [19] or the altered physiology in bees suggesting a decrease in lifespan [8]. Here, we link the decreased lifespan of individual bees due to *V. destructor* infestation to colonies losses in at least some circumstances.

Mattila et al. [12] showed an increase in bee longevity between August and the beginning of November, which fully agrees with our findings: the number of capped brood cells decreased drastically between August and November, while at the same time, the lifespan of the bees increased indicating the transition of short-lived to long-lived winter populations [20]. When low infestation of *V. destructor* occurred earlier in the period of winter bee transition, lifespan of the bees increased and consequently the winter survival of the colonies increased, which supports previous findings by Delaplane and Hood [13] and Currie and Gatien [14].

In our study, however, mean lifespan (estimated by bee survival at 100 days) was longer during the winter in 2005/2006, compared to the winter of 2006/2007. A variety of stress-related factors such as winter temperatures or foraging conditions in autumn, could have contributed to the variation in lifespan between years. The much shorter lifespan for bees during the winter 2006/2007 at least suggests that bees were more active during this winter. Possibly due to the observed rearing of brood, as long lifespan is inhibited by brood pheromones [21] and reduced by brood rearing activities depleting body reserves [20], [22]. This shorter lifespan, however, may have been less problematic due to the earlier start of spring [8] illustrated by the high number of brood cells in April 2007 compared to the year before.

Although winter temperature was not included as a replicated treatment, we observed that mean lifespan (estimated by bee survival at 100 days) was longer during the colder winter in 2005/2006, compared to the relatively mild winter of 2006/2007. Mean longevity in the study of Mattila et al. [12] was longer than in our study, for comparison: on October 6^th^ longevity ranged between 125–150 days in the study of Mattila et al. [12], while in our study on this date mean longevity was 62 days for 2006/2007 and 93 for 2005/2006 (calculated using the method described in Matilla et al. [12]). Mattila et al. [12] performed their experiments in the south of Manitoba, Canada, which has approximately the same latitude as The Netherlands, but has a continental climate characterized by large annual amplitudes in temperature instead of an oceanic climate as in our site with narrow annual temperature amplitudes. The even lower winter temperatures in Canada compared to the Netherlands can maybe explain the longer lifespan of the Canadian bees. We therefore hypothesize that the negative effect of *V. destructor* (i.e., shortened lifespan of winter bees and possible colony loss) is larger under colder winter conditions.

Colony survival, measured by the number of frames with bees occupied in April, was highest with treatment against *V. destructor* applied in July, due to the longest lifespan of the bees (bee survival at 100 days) in autumn for these colonies. Delaplane and Hood [13] also studied the effects of timing of acaricide treatment (with Apistan) on honeybee colonies parasitized by *V. destructor*, where type of one-story hives, colony sizes and amounts of brood were comparable to our experiment. They found that colony survival and colony size, measured in December, was higher by acaricide treatment in August (in contrast to treatment in June or October). In their study, colonies treated in October resulted in unacceptably high bee mortality in December. Mite fall before treatment of these colonies was 145±30 mites per 18±5 h, which was much higher than mite fall in November in our study (max. 32±11 mites per 24 h; mite fall in December in our study was not representative for ‘natural’ mite fall due to the acaricide treatment in this month). In our study, however, at this relatively low level of mite fall, colony loss already occurred. Our late treatment (September) did not show an increase in colony size (in April), and nor did theirs (October, resulted in a 45% decline in colony size in December). Acaricide treatments to kill *V. destructor* in late autumn may thus fail to prevent losses of colonies because many of the adult bees are no longer able to survive until spring [8].

We manipulated the level of *V. destructor* infestation by using acaricide treatments at different moments. This acaricide treatment with Thymovar® was effective because mitefall was indeed increased during the month the acaricide treatment was applied. The pattern of mite fall directly after the acaricide treatment for the different moments (Figure 3) confirms with the expected infestation level after the month of treatment (Figure 2). The efficacy of Thymovar® as an acaricide has been shown before: 72% for one-story and 94% for two-story colonies [23], or 97% for one-story colonies with low amount of brood [24]. Although mite fall was reduced after the acaricide treatment in July, August or September, it was not as much reduced as after the treatment using oxalic acid in December. Oxalic acid however only affects mites in the phoretic phase, which is the predominant phase during winter when brood rearing has stopped or is reduced [25], [26]. This is supported by the slightly higher mite fall during winter 2006/2007 compared to 2005/2006, and the most likely higher amount of reared brood (assumed during winter 2005/2006, not measured).

Previous studies showed that *V. destructor* infestation reduces the body weight and protein content of individual bees, which shortens their lifespan [6]–[8]. Our study supports these findings and shows the relation between decreased lifespan of individual bees and increased colony losses. Additionally, colonies treated earlier in the season had reduced *V. destructor* infestation before the development of winter bees resulting in longer bee lifespan and higher colony survival after winter (Figure 7).

This study contributes to theory about the multiple causes for the recent elevated colony losses in honey bees. Our study shows that high *V. destructor* infestation during the transition to winter bees can cause these colonies losses due to decreased lifespan of winter bees. We can expect that other environmental stresses, such as pesticides, other pathogens, decreased food availability, or reduced diversity of this food [1], [3], [27], in combination with *V. destructor* will further reduce lifespan of bees and increase colony losses during and after winter.

## Supporting Information

Figure S1 **Mean survivorship curves for cohorts of bees marked in 2005/2006 (left) and 2006/2007 (right).** Cohorts of bees were marked at 14-day intervals for each acaricide treatment: July (open diamonds), August (grey squares), September (dark grey triangles), and not treated at all (black circles). Each line shows the mean survival of 1 to 4 cohorts. During the winter of 2005/2006, actual counts of marked bees were suspended due to cold temperatures; mortality was assumed to be constant for that period.
(TIF)

Figure S2 **Cumulative survival curves for 2005/2006 (top) and 2006/2007 (bottom), per acaricide treatment (left), and for the marking date of the cohorts (right).** Cumulative survival curves were calculated from the Cox Proportional Hazards Models for cohorts of bees marked. For the survival analysis, we had 6398 uncensored cases and 346 censored cases for 2005/2006, and 8458 uncensored cases and 547 censored cases for 2006/2007. During the winter of 2005/2006, actual counts of marked bees were suspended due to cold temperatures; mortality was assumed to be constant for that period.
(TIF)
