# Supplementary figures and images for: Winter Survival of Individual Honey Bees and Honey Bee Colonies Depends on Level of Varroa destructor Infestation
Source: PLoS One. 2012 Apr 27;7(4):e36285. doi: 10.1371/journal.pone.0036285 (PMC3338694; doi:10.1371/journal.pone.0036285)

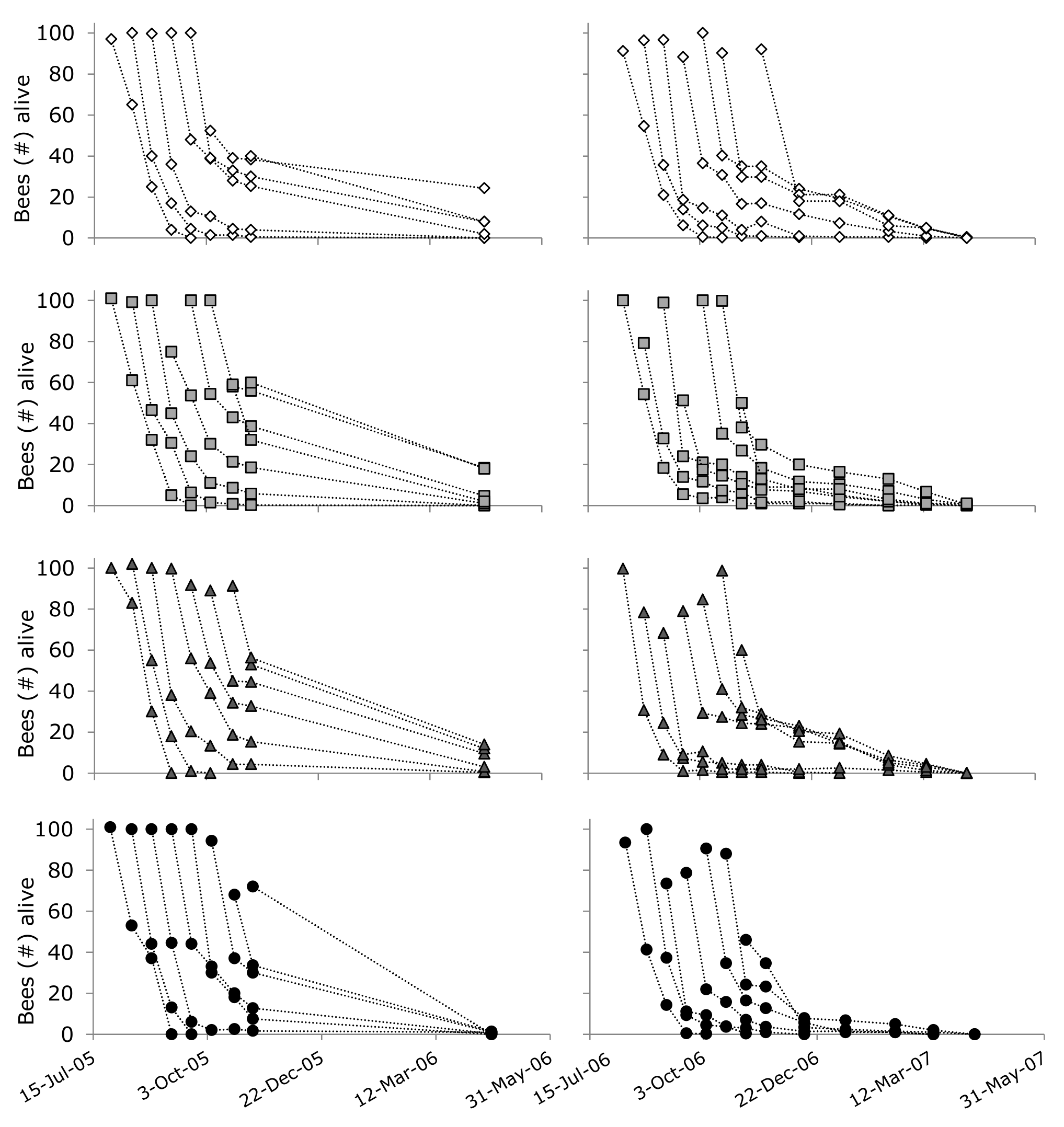

Supplement: Figure S1 — Mean survivorship curves for cohorts of bees marked in 2005/2006 (left) and 2006/2007 (right). Cohorts of bees were marked at 14-day intervals for each acaricide treatment: July (open diamonds), August (grey squares), September (dark grey triangles), and not treated at all (black circles). Each line shows the mean survival of 1 to 4 cohorts. During the winter of 2005/2006, actual counts of marked bees were suspended due to cold temperatures; mortality was assumed to be constant for that period. (TIF) [file pone.0036285.s001.tif]

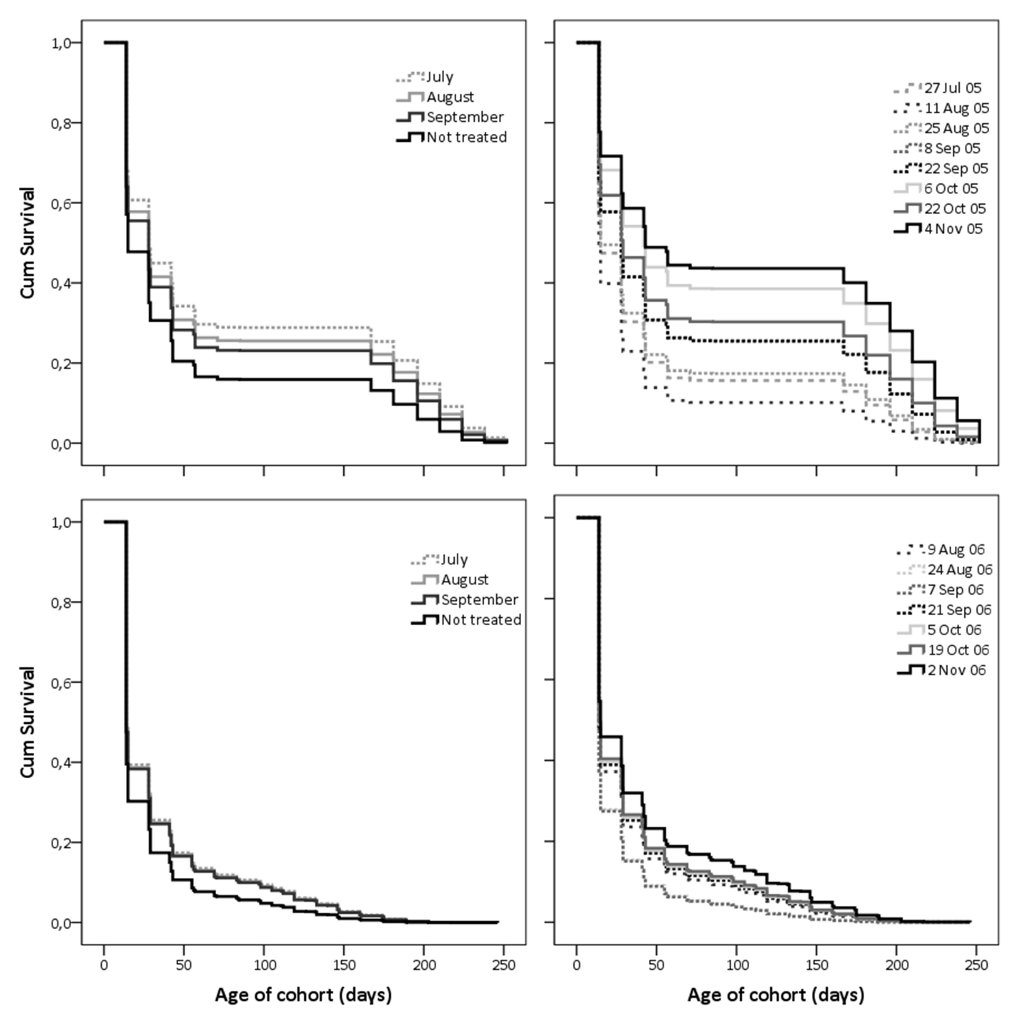

Supplement: Figure S2 — Cumulative survival curves for 2005/2006 (top) and 2006/2007 (bottom), per acaricide treatment (left), and for the marking date of the cohorts (right). Cumulative survival curves were calculated from the Cox Proportional Hazards Models for cohorts of bees marked. For the survival analysis, we had 6398 uncensored cases and 346 censored cases for 2005/2006, and 8458 uncensored cases and 547 censored cases for 2006/2007. During the winter of 2005/2006, actual counts of marked bees were suspended due to cold temperatures; mortality was assumed to be constant for that period. (TIF) [file pone.0036285.s002.tif]
